# Moving toward a precise nutrition: preferential loading of seeds with essential nutrients over non-essential toxic elements

**DOI:** 10.3389/fpls.2014.00051

**Published:** 2014-02-20

**Authors:** Mather A. Khan, Norma Castro-Guerrero, David G. Mendoza-Cozatl

**Affiliations:** Division of Plant Sciences, Christopher S. Bond Life Sciences Center, University of MissouriColumbia, MO, USA

**Keywords:** food security, heavy metals, long distance transport, seed loading, mineral nutrition

## Abstract

Plants and seeds are the main source of essential nutrients for humans and livestock. Many advances have recently been made in understanding the molecular mechanisms by which plants take up and accumulate micronutrients such as iron, zinc, copper and manganese. Some of these mechanisms, however, also facilitate the accumulation of non-essential toxic elements such as cadmium (Cd) and arsenic (As). In humans, Cd and As intake has been associated with multiple disorders including kidney failure, diabetes, cancer and mental health issues. Recent studies have shown that some transporters can discriminate between essential metals and non-essential elements. Furthermore, sequestration of non-essential elements in roots has been described in several plant species as a key process limiting the translocation of non-essential elements to aboveground edible tissues, including seeds. Increasing the concentration of bioavailable micronutrients (biofortification) in grains while lowering the accumulation of non-essential elements will likely require the concerted action of several transporters. This review discusses the most recent advances on mineral nutrition that could be used to preferentially enrich seeds with micronutrients and also illustrates how precision breeding and transport engineering could be used to enhance the nutritional value of crops by re-routing essential and non-essential elements to separate sink tissues (roots and seeds).

## INTRODUCTION

Plants and seeds are the main dietary source of micronutrients (i.e., zinc, copper, manganese, and iron). Over the last 50 years, the production of cereals has tripled and the demand is expected to rise due to the constantly increasing population (Gregory and George, 2011). Any effort devoted to increase yield has to ensure that the nutritional value of seeds and grains is retained or, preferably, improved. The consumption of grains with low quantity of micronutrients has been associated with mineral deficiencies in humans (Burchi et al., 2011; Murgia et al., 2012). More than 2 billion people are affected by at least one type of micronutrient deficiency (White and Broadley, 2009; Waters and Sankaran, 2011). Another concern that could impact food security is the consumption of non-essential elements such as cadmium (Cd) and arsenic (As) through contaminated food. More than 80% of Cd intake in humans comes from cereals and vegetables (Olsson et al., 2002; Egan et al., 2007). Natural occurrence of As in groundwater used for irrigation has been found around the world (Nordstrom, 2002; Rodriguez-Lado et al., 2013) potentially exposing more than 100 million people to As. Chronic exposure to low levels of Cd and As has been associated with cancer, diabetes, renal failure and neurological disorders (Satarug et al., 2010; Cho et al., 2013; Clemens et al., 2013).

**FIGURE 1:**
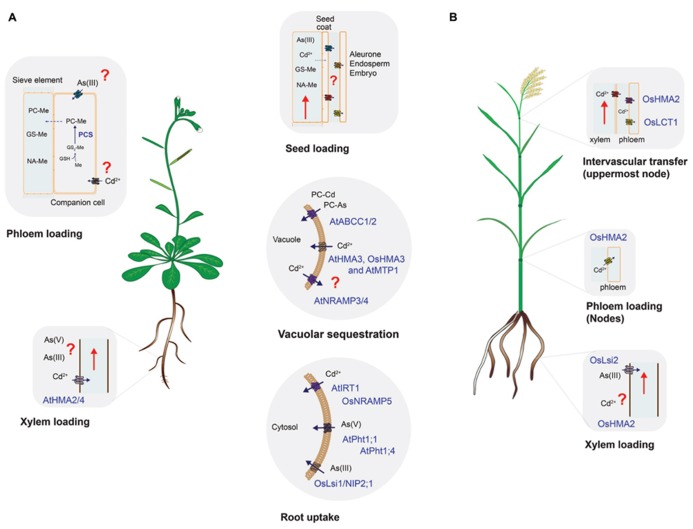
** Uptake and distribution of cadmium and arsenic in (A) *Arabidopsis* and (B) rice**. Processes conserved in both species (root uptake, vacuolar sequestration, and seed loading) are shown in the middle panels. Species-specific transporters are denoted by At (*Arabidopsis*) or Os (rice). Processes where information is scarce, absent or that require further research are labeled with red question marks. The embryo and the maternal tissues (seed coat) are symplastically isolated, thus molecules have to be unloaded from the phloem to later be taken up by embryo-related tissues. Unloading and uptake of molecules in reproductive tissues is thought to be mediated by specific transporters but the identity of these proteins has not been established. Only proteins characterized *in vitro* and *in planta *are shown. For simplicity, co-substrates such as H^+^ or ATP-Mg were omitted. GSH, glutathione; Me, metal(loid) such as Cd or As(III); PCS, phytochelatin synthase; PC–Me, phytochelatin–metal(loid) complex; GS–Me, glutathione–metal(loid) complex; NA–Me, NA–metal complex.

Uptake and distribution of nutrients from the soil and within the plant is a dynamic process driven by root uptake transporters, root-to-shoot translocation (xylem transport) and source-to-sink transport through the phloem, which includes seed loading (**Figure 1** and **Table 1**) (Mendoza-Cozatl et al., 2011; Waters and Sankaran, 2011; Sinclair and Kramer, 2012). Non-essential elements like As and Cd are taken up by plants and distributed between plant tissues by the same transporters that mobilize nutrients such as Fe^2^^+^, Zn^2^^+^, Mn^2^^+^, or PO_4_^2^^-^. Therefore, each of the steps required to move nutrients from the soil and into seeds (root uptake, translocation, xylem and phloem transport) represent an opportunity to increase the specificity towards essential nutrients and re-route non-essential elements to non-edible parts of the plant (**Figure 1**). Understanding the molecular mechanisms of each transport process and the contribution of different transporters to the overall allocation of essential and non-essential elements within plant tissues will help in developing crops that yield grains of higher nutritional value with lower content of non-essential elements. This review discusses the most recent discoveries in plant nutrition that could help achieving the goal of safe nutritional enrichment by means of precision breeding or transport engineering (Collard and Mackill, 2008; Nour-Eldin and Halkier, 2013).

**Table 1:** Transporters mediating the uptake and mobilization of arsenic or cadmium (discussed in this review).

Transporter	Localization	Function/substrate	Reference
AtIRT1	Root (PM)	Uptake of Fe, Zn, Mn and Cd	Rogers et al. (2000)
OsHMA3	Root (Vac)	Cd sequestration in root vacuoles	Ueno et al. (2010)
AhHMA3	Root/shoot (Vac)	Zn sequestration in root vacuoles	Becher et al. (2004)
AtHMA3	Vascular tissues and root apex (Vac)	Transport of Zn, Cd, Co and Pb	Morel et al. (2009)
OsNRAMP5	Root (PM)	Uptake of Mn and Cd	Sasaki et al. (2012)
AtPht1;1/1;4	Root hair cells, root cap (PM)	Uptake of PO_4_^3^^-^ and arsenate (As^V^)	Shin et al. (2004)
OsNip2;1 (OsLsi1)	Roots, exodermis, and endodermis (PM)	Uptake of arsenate (As^III^)	Ma et al. (2008)
OsLsi2	Roots, exodermis, and endodermis (PM)	Efflux of arsenite (As^III^)	Ma et al. (2008)
AtABCC1/2	Root/shoot (Vac)	Arsenite–PC transporter	Song et al. (2010b)
AtHMA2/4	Roots, vascular tissue, and leaves (PM)	Xylem loading of Cd/Zn	Hussain et al. (2004)
OsHMA9	Vascular bundles and anthers (PM)	Cu, Zn and Cd detoxification	Lee et al. (2007)
OsHMA2	Roots and vascular bundles (PM)	Zn/Cd delivery to developing tissues	Yamaji et al. (2013)
AtPBR2	Roots, epidermal cells, and xylem (PM)	Zn homeostasis and Cd transport	Song et al. (2004),2010a
OsNRAMP1	Root and shoot (PM)	Cd uptake	Takahashi et al. (2011)
AtMTP1	Roots and leaves (Vac)	Zn and Cd transporter	Desbrosses-Fonrouge et al. (2005)
AtZIF1	Roots and leaves (Vac)	Mobilization of nicotianamine	Haydon and Cobbett (2007)
AtNRAMP3/4	Vascular bundles, roots, and leaves (Vac)	Fe efflux transporter	Lanquar et al. (2005); Thomine et al. (2003)

*The first two letters denote the organism of origin: At, *Arabidopsis thaliana; *Ah, *Arabidopsis halleri*; Os, *Oryza sativa.* Subcellular localization is shown in parenthesis: PM, plasma membrane; Vac, vacuole (tonoplast). See also **Figure 1** and text for further details.*

## ROOT UPTAKE, VACUOLAR SEQUESTRATION, AND ROOT-TO-SHOOT TRANSLOCATION OF Cd AND As

Root uptake represents the first step where selectivity towards essential elements can be improved and evidence suggest that it is possible to increase the selectivity of transporters towards essential elements. In *Arabidopsis*, uptake of Fe^2^^+^, Zn^2^^+^, Mn^2^^+^, and Cd^2^^+^ is mediated by the ZIP family of transporters (Palmer and Guerinot, 2009; Mendoza-Cozatl et al., 2011). IRT1 is one of the best characterized members of this family and despite its broad specificity, previous studies have shown that changes in specific amino acid residues can modify its substrate specificity (Rogers et al., 2000). These studies have shown that discrimination between Fe^2^^+^/Mn^2^^+^ against Cd^2^^+^ is feasible. Selectivity of Zn^2^^+^ over Cd^2^^+^ is more challenging due to the highly similar electron configuration between these two elements. Selectivity of Zn^2^^+^ over Cd^2^^+^, however, has been demonstrated for mammalian ZnT transporters and bacterial P-type ATPases (Hoch et al., 2012). Conversely, selectivity of Cd^2^^+^ over Zn^2^^+^ has been found in the rice vacuolar P1B-type ATPase OsHMA3 and this gene has been identified as a major determinant limiting Cd accumulation in rice grains (see below; Ueno et al., 2010). The molecular basis and amino acid residues required for this metal selectivity have not been determined and could potentially be used in other crop species to limit the accumulation of Cd in seeds. In rice, the natural resistance-associated macrophage protein 5 (OsNRAMP5) has been identified as the main Mn^2^^+^ and Cd^2^^+^ transporter at the root level (**Figure 1** and **Table 1**; Sasaki et al., 2012). OsIRT1 and OsIRT2 also mobilize Fe^2^^+^, Zn^2^^+^, and Cd^2^^+^ into roots but these transporters seem to play a minor role in Cd uptake compared to OsNRAMP5 (Sasaki et al., 2012). Substrate specificity studies could help identifying OsNRAMP5 natural variants with preference for Mn^2^^+^ over Cd^2^^+^ that can be introgressed into high-yield varieties of rice.

In contrast to Cd, As can occur in soils in several oxidation states, being arsenate (As^V^) and arsenite (As^III^) the most common ones (Ma et al., 2008; Ali et al., 2009; Zhao et al., 2009). In *Arabidopsis*, As^V^ is taken up from aerobic soils by the phosphate transporters AtPht1;1 and 1;4 (Shin et al., 2004). The striking similarities between phosphate and arsenate in terms of p*K*a values, charged oxygen atoms and thermochemical radii differing only by 4% make a substrate specificity approach very challenging (Elias et al., 2012). Some bacterial phosphate binding proteins are able to discriminate between phosphate and arsenate and the structural basis for this discrimination has recently been established (Elias et al., 2012). Similar structural studies with plant transporters could help engineering phosphate transporters with high selectivity for phosphate over As^V^. Once inside the cells, As^V^ is quickly reduced to As^III^, which is the predominant form of inorganic arsenic in plant cells (Ali et al., 2009; Zhao et al., 2009). Arsenite also occurs mostly in anaerobic soils and can be taken up by plants thus affecting flooded crops like rice. OsNIP2;1, a member of the Nod26-like major intrinsic protein (NIP) has been identified as a major pathway for As^III^ uptake into rice roots (Ma et al., 2008; Zhao et al., 2009). The physiological function of OsNIP2;1, also known as Lsi1, is silicon uptake. Lsi2 on the other hand, functions as a silicon and As^III^ exporter and both Lsi1 and Lsi2 are the main transporters controlling As^III^ uptake and translocation to shoots (**Figure 1** and **Table 1**). Recently, there have been some efforts to identify amino acid residues affecting the substrate selectivity of OsNIP2;1/Lsi1 but so far no selectivity of essential nutrients over As^III^ has been found (Mitani-Ueno et al., 2011).

Sequestration in root vacuoles has been demonstrated as the major process limiting the translocation of As and Cd to shoots and seeds (Ueno et al., 2010; Kopittke et al., 2013). Similar to Cd^2^^+^, As^III^ has a strong affinity for thiol-containing molecules such as cysteine, glutathione and phytochelatins (PCs). PCs are glutathione-derived peptides synthesized in response to As^III^ or Cd^2^^+^ exposure (Mendoza-Cozatl et al., 2005). PC–metal(loid) complexes are transported into vacuoles by ATP-binding cassette transporters and, in *Arabidopsis*, ABCC1 and ABCC2 have been identified as the main transporters mediating PC uptake into vacuoles (Song et al., 2010b). Orthologs of AtABCC1 have been identified in grasses including rice, maize and barley and kinetic analysis using vacuoles isolated from barley suggest that the transport mechanism is conserved across species (Song et al., 2013). Furthermore, a recent analysis using six rice varieties showing low and high accumulation of arsenic in grains suggest that PCs play a key role trapping inorganic As in roots, limiting the transfer of As to shoots and grains (Batista et al., 2013). PCs are usually considered as a mechanism to detoxify non-essential elements; however, there is evidence to suggest that PCs also play a role in the homeostasis of essential metals such as Zn and Mn (Tennstedt et al., 2009; Song et al., 2013). Therefore, more studies are needed to explore whether increasing vacuolar sequestration of PCs in roots could have a negative impact on the content of essential elements in aboveground edible tissues.

P1B-type ATPases have also been shown to play a key role sequestering Cd in root vacuoles (Ueno et al., 2010). OsHMA3, a vacuolar P1B-type ATPase was found to be responsible for 85.6% of the variance in Cd content between low- and high-cadmium accumulation varieties of rice (Ueno et al., 2010). OsHMA3 sequesters Cd in root vacuoles, thus preventing it from reaching shoots and grains. Of particular interest is the fact that OsHMA3 seems to be highly specific for Cd while the *Arabidopsis halleri* HMA3 has preference for Zn and the *A. thaliana* HMA3 shows broad substrate specificity being able to transport Co, Pb, Cd, and Zn (Becher et al., 2004; Morel et al., 2009). Structural studies using OsHMA3, AhHMA3, and AtHMA3 could help identifying which residues give the high specificity of OsHMA3 for Cd over essential elements such as Zn or Mn.

Xylem loading is the next step where translocation of non-essential elements can be blocked allowing only essential elements to reach leaves and seeds. In *Arabidopsis* Cd and Zn are loaded into the xylem by two P1B-type ATPases, HMA2, and HMA4 (Hussain et al., 2004). It would be interesting to determine whether it is possible to alter the selectivity of HMA2 and 4 to favor the translocation of Zn over Cd. PCR2 is another *Arabidopsis* protein that has been implicated in the long-distance transport of Cd and Zn (Song et al., 2004, 2010a). However, PCR2 expression is not restricted to xylem parenchyma suggesting that PCR2 may have other roles beside root-to-shoot translocation of Cd and Zn (Song et al., 2004, 2010a). Less information is known about the HMA family in rice, OsHMA2 has been proposed to mediate the loading of Cd and Zn into the xylem (Takahashi et al., 2012), together with OsHMA9 and proteins from the NRAMP family of transporters (OsNRAMP1; Lee et al., 2007; Takahashi et al., 2011). OsHMA5 has also been localized in root pericycle cells and xylem but OsHMA5 seems to be a copper-specific transporter with little or no effect on the accumulation of Fe, Mn, or Zn in rice tissues (Deng et al., 2013).

Metal(loid)–ligand chemistry is another process that is key to understand how elements are mobilized throughout the plant. For instance, Fe and Cd are taken up by IRT1, but once inside the cell, Fe and Cd will form complexes with different ligands; iron will prefer oxygen-containing molecules such as citrate and nicotianamine while Cd will bind to thiol- and nitrogen-containing compounds such as glutathione, PCs and histidine (Meda et al., 2007; Mendoza-Cozatl et al., 2008; Rellán-Álvarez et al., 2008). This metal–ligand interaction explains why Cd and Zn, but not Fe, compete for the same ligands and transporters.

Inorganic arsenic can reach the xylem as As^V^ or As^III^ but fluorescence-X-ray absorption near-edge spectroscopy (fluorescence-XANES) has recently shown that As^III^ is the predominant form of arsenic in plant cells (Kopittke et al., 2013). Similar to Cd, As^III^ has a strong affinity for thiol groups and, consequently, most of the As^III^ found in the cortex and stele was identified as As(III)–thiol complexes (Kopittke et al., 2013). In rice, Lsi2 has been identified as the transporter mediating As^III^ loading into the xylem (Ma et al., 2008). In *Arabidopsis*, it is not clear which transporter mediates this process but transporters of the NIP family are possible candidates that are currently being evaluated (Ali et al., 2009; Kamiya et al., 2009).

### INTRACELLULAR TRANSPORT AND PHLOEM LOADING

In contrast to natural As and Cd hyperaccumulators, *Arabidopsis*, rice and wheat accumulate only a minor fraction of As and Cd in leaves. In *Arabidopsis*, MTP1 has been described as a Cd/Zn vacuolar transporter and structure–function analyses suggest that it is possible to increase the selectivity of AtMTP1 towards Zn (Desbrosses-Fonrouge et al., 2005; Kawachi et al., 2012; Podar et al., 2012). OsMTP8.1 has been described as a vacuolar Mn-specific transporter in rice but its role on Cd uptake has not been evaluated (Chen et al., 2013). ZIF1 is a tonoplast-localized transporter that mobilizes nicotianamine into *Arabidopsis* vacuoles thus affecting Fe and Zn homeostasis (Haydon et al., 2012). Deletion of ZIF1 leads to Cd hypersensitivity in *Arabidopsis* but it is not clear whether this sensitivity is the result of an impaired Fe/Zn homeostasis or whether ZIF1-mediated transport of nicotianamine plays a role in sequestering Cd in shoot vacuoles. *Arabidopsis* NRAMP3 and 4 are transporters that export Fe from vacuoles (Lanquar et al., 2005). When expressed in yeast, NRAMP3 and 4 can mobilize Cd and it has recently been shown that photosynthesis in the *Arabidopsis* *nramp3 nramp4 *double mutant is particularly sensitive to Cd (Molins et al., 2013). NRAMP proteins, like ZIP transporters, have broad substrate specificity and more structural analyses are needed to determine how NRAMP proteins could be used to restrict the accumulation of non-essential elements while preserving the homeostasis of essential metals.

The low accumulation of As and Cd in shoots observed in non-hyperaccumulator species was thought to be solely the result of limited translocation to shoots but recent evidence suggest that low-retention in leaves together with phloem-mediated transport to roots could play an important role re-allocating non-essential elements from shoots to roots (Mendoza-Cozatl et al., 2008, 2011; Ye et al., 2010). Nutrients and non-essential molecules are loaded into seeds through the phloem, which is a vascular system made up of two highly specialized cells, companion cells and sieve elements (Moore et al., 2013; Slewinski et al., 2013). Companion cells transfer molecules into sieve elements for long-distance transport, thus transporters expressed in companion cells are critical proteins that could impact phloem sap and seed composition. In contrast to xylem transport, phloem loading of micronutrients and non-essential elements remains largely unexplored. However, the phloem plays a key role in delivering nutrients to developing seeds, where xylem transport is limited. *Arabidopsis* YSL1 and YSL3 are nicotianamine–metal transporters required to mobilize Mn, Zn, Cu and Fe out of senescing leaves but their role in Cd mobilization, if any, has not been evaluated. Similarly, rice OsYSL16 has been characterized as a Cu–NA transporter while OsYSL6 is required to detoxify excess of Mn but neither OsYSL16 or OsYSL6 seem to participate in Cd mobilization to developing grains (Sasaki et al., 2011; Chen et al., 2013).

Phloem loading mechanisms may vary across species and in the case of rice, xylem-to-phloem transfer, particularly at the node connected to the flag leaf and panicle, appears to be the major route for Cd loading into the phloem. Recently, OsLCT1 was identified as a strong candidate mediating the intervascular transfer of Cd into the phloem. OsLCT1 is strongly expressed in nodes during grain ripening and RNAi-mediated knockdown of OsLCT1 resulted in 50% less Cd in grains without affecting the concentration of essential micronutrients or grain yield (Uraguchi et al., 2011). OsHMA2, besides being expressed in roots, is also expressed in the nodes (phloem) at reproductive stage and insertional mutants showed less Cd and Mn in the upper nodes compared to wild-type (Yamaji et al., 2013). Thus, OsLCT1 and OsHMA2 could be the target for further manipulation to limit the translocation of Cd into rice grains.

### TOWARD A PRECISE NUTRITION

Several reviews have recently discussed how QTL mapping, GWAS, transgenic approaches and conventional breeding have successfully been used to increase the concentration of micronutrients in seeds (White and Broadley, 2005, 2009; Sperotto et al., 2012). From these studies it is clear that several processes regulating the movement of elements into seeds are still largely unknown and that the mechanisms mediating long-distance transport of nutrients vary widely across species. Moreover, because non-essential elements such as As and Cd use the transport systems for essential nutrients to move within the plant, efforts to increase the content of micronutrients in crops could also increase the concentration of toxic elements in seeds. This is a serious threat to food security particularly in places where the occurrence of non-essential toxic elements in groundwater used for irrigation is above safety limits.

A successful precision breeding or transport engineering approach to produce crops able to accumulate micronutrients over non-essential metals should consider: (i) high substrate selectivity of transporters for essential metals, (ii) tissue-specific expression and subcellular localization, (iii) the developmental stage at which the transporter is needed, and (iv) the need of ligand molecules in sink tissues to keep micronutrients bioavailable and prevent toxicity. Research suggests that successful approaches will likely require the simultaneous modification of more than one step in different tissues. Cross-species studies of transporters of the same family, crystallography and site-directed mutagenesis have shown to be extremely useful to identify amino acid residues critical to enhance the selectivity of transporters (Becher et al., 2004; Courbot et al., 2007; Zimmermann et al., 2009). The lower cost of next-generation sequencing together with natural variant accessions available for some crops makes feasible the identification of transporters that can later be introgressed into high yield varieties to obtain safer and more nutritious grains. While transgenic approaches have a faster turnaround compared to traditional breeding, other technologies such as precision breeding or genome editing are non-transgenic alternatives that could achieve similar results in comparable time frames. Also, the development of tissue-specific translatome analysis in *Arabidopsis* offers the opportunity, for the first time, to study the regulation of transporters at tissue-specific level (Mustroph et al., 2009). Phloem unloading and re-uptake of nutrients by seed tissues is the last barrier where the accumulation of non-essential metals in seeds could be blocked. The identity and regulation of transporters mediating phloem unloading and seed loading are largely unknown and phloem-specific translatome analysis in seed loading tissues could help identifying such transporters. Translatome analyses are also currently being established in crop plants (Mendoza-Cozatl and Stacey, unpublished) and will likely provide more details about the mechanisms mediating the mobilization of nutrients and toxic elements within the plant and ultimately into seeds.

The urgent need of providing more nutritious food to a rapidly growing population is challenging considering environmental issues such as climate change, contamination of soil and water, and land available, but sustainable solutions to these global challenges are more likely to come from cross-disciplinary approaches between farmers, breeders, biologists, geneticists and bioengineers.

## Conflict of Interest Statement

The authors declare that the research was conducted in the absence of any commercial or financial relationships that could be construed as a potential conflict of interest.
